# Association between inflammatory score, healthy lifestyle, and cardiovascular disease: a national cohort study

**DOI:** 10.3389/fnut.2025.1534458

**Published:** 2025-02-20

**Authors:** Han-Qing Zhao, Jia-Le Lv, Yuan-Zhi Gao, Bo Hu, Zong-Da Du, Yan Wang, Mei-Lin Wang, Meng-Di Hou, Fang Li, Xue Xing, Ming-Hui Sun

**Affiliations:** ^1^Hunan Provincial Center for Disease Control and Prevention, Changsha, China; ^2^The Third Affiliated Hospital of Jinzhou Medical University, Jinzhou, China; ^3^Department of Clinical Epidemiology, Shengjing Hospital of China Medical University, Shenyang, China; ^4^Clinical Research Center, Shengjing Hospital of China Medical University, Shenyang, China; ^5^Liaoning Provincial Center for Disease Control and Prevention, Shenyang, China; ^6^The Fourth Affiliated Hospital of Harbin Medical University, Harbin, China; ^7^Department of Pathology, Shengjing Hospital of China Medical University, Shenyang, China

**Keywords:** cardiovascular disease, China, cohort study, healthy lifestyle, inflammatory score

## Abstract

**Background:**

The inflammation score is currently regarded as a reliable composite index for comprehensive assessment of inflammatory status. However, the relationship between inflammation score and cardiovascular disease (CVD) is unclear. Thus, we aimed to explore the association of inflammatory score with CVD, as well as to evaluate whether adhering to a healthy lifestyle could alleviate this association.

**Methods:**

We analyzed 6,164 participants aged ≥45 years who entered a prospective cohort study of the China Longitudinal Study of Health and Retirement (CHARLS) between 2011 and 2012 and were followed up for CVD incidence untill 2018. The inflammatory score was measured by summing of the Z-scores for C-reactive protein and white blood cell count at baseline. The healthy lifestyle score was calculated by four factors, smoking status, alcohol consumption, body mass index, and sleep duration. Cox proportional hazard models were utilized to calculate the hazard ratios (HRs) and 95% confidence intervals (CIs) for the incidence of CVD.

**Results:**

During the 7-year follow-up period, there were 761 incident cases of CVD. Compared with the lowest tertiles, the highest inflammatory score was associated with an elevated risk of CVD (HR = 1.25, 95% CI = 1.04–1.49). Compared to the unhealthy lifestyle, participants adhered to a healthy lifestyle was inversely associated with CVD risk (HR = 0.74, 95% CI = 0.60–0.93). Of note, when participants adhered to a healthy lifestyle, the higher inflammatory score was no longer significantly correlated with CVD risk (HR = 1.00, 95% CI = 0.76–1.34). Additionally, a multiplicative interaction was detected between inflammatory score and healthy lifestyle score for CVD risk (*p* interaction <0.05).

**Conclusion:**

The inflammation score was associated with higher risk of CVD incidence, but adherence to a healthy lifestyle may mitigate the adverse association of inflammation score and CVD among the middle-aged and older participants.

## Introduction

Cardiovascular disease (CVD) predominantly denote disorders resulting from structural or functional anomalies of the heart and blood vessels (1). These diseases cover a wide range of specific conditions, including, but not restricted to, coronary artery disease, hypertension, and arrhythmias. Globally, CVD represents the predominant cause of disability and death among adults (1). Over the past 30 years, the global prevalence of CVD cases has escalated markedly from 271 million in 1990 to 523 million in 2019, representing an increase of 92.3% (2). Concurrently, the number of CVD-attributable deaths increased from 12.1 million in 1990 to 18.6 million in 2019, an increase of 53.7% (2). Therefore, current strategies for identifying high-risk individuals need to be strengthened and the widespread use of biomarkers for CVD assessment is warranted to further reduce the risk of CVD incidence. A mounting body of epidemiological evidence suggests that inflammatory markers might be correlated with the risk of this disease (3–6).

Inflammation has a crucial impact in the pathogenesis and progression of CVD, because it promotes atherosclerotic plaque progression and endothelial cell damage (7). Nevertheless, these existing studies investigating the association of inflammatory markers with CVD remain lacking and mainly focus on individual inflammatory markers. For example, some research have indicated that inflammatory markers existing in the human body, such as white blood cell (WBC) count and C-reactive protein (CRP) and other inflammatory indicators, may have adverse impacts on the risk of CVD (8–10). A combination of inflammatory markers (CRP and WBC) can make up inflammatory score (calculated through the addition of the Z-scores of WBC and CRP). The inflammation score is currently regarded as a reliable composite index for comprehensive assessment of inflammatory status. The inflammatory score has the advantages of being more reliable, systematic, and comprehensive in reflecting the inflammatory burden, and it is worthy of being recommended for use in future studies (11, 12). However, as far as we know, there is currently no prospective study on the relationship between the combined exposure of multiple inflammatory markers and CVD has been conducted.

Meanwhile, several lifestyle-related factors, namely obesity, alcohol consumption, smoking, and sleep patterns, have been shown to be correlated with CVD risk (13–16). As these lifestyle factors tend to coexist, emerging studies have started to utilize lifestyle indices to evaluate the impacts of the combinations of individual lifestyle factors on CVD incidence (17, 18). For example, results from two prospective cohort studies suggest that adherence an overall healthy lifestyle, including never smoking, not drinking excessively, engaging in high-level physical activity, and maintaining a high-quality diet may decrease the risk of CVD (18). Furthermore, a longitudinal cohort study with data from 96,364 participants confirmed an inverse relationship between an overall healthy lifestyle score and CVD risk (19). These findings suggested that, from an individual point of view, integrating modifiable healthy lifestyle interventions into national health management was of great significance. Nevertheless, it is unclear whether adhering to an overall healthy lifestyle can decrease the risk of CVD incidence in participants exposed to higher inflammation score.

Hence, to address the aforementioned knowledge gaps, based on the longitudinal study of the China Health and Retirement Longitudinal Study (CHARLS), we assessed the association between CVD and inflammatory score and further evaluated whether healthy lifestyle behaviors modified this association.

## Methods

### Study population and data source

We utilized the data of the CHARLS. The CHARLS was a population-based longitudinal cohort study that is prospective and nationally representative. Previously, details regarding the study methods and characteristics of the included participants have been provided (1, 20). The CHARLS study encompassed 450 urban and rural communities within 28 provinces in China. During the baseline survey, 17,708 individuals took part in face-to-face interviews, with 80.5% of the participants responded to our survey Trained interviewers used standardized electronic questionnaire to gather information regarding participants’ socio-demographic characteristics (such as year of birth, gender, and income level), lifestyle behaviors (such as dietary frequency, sleep duration, social situation, smoking, and drinking stutus), and insurance participation status. The baseline survey of CHARLS was implemented in 2011–2012. Comprehensive follow-ups similar to baseline examinations were conducted on the participants of this study every 2 years.

**Figure 1:**
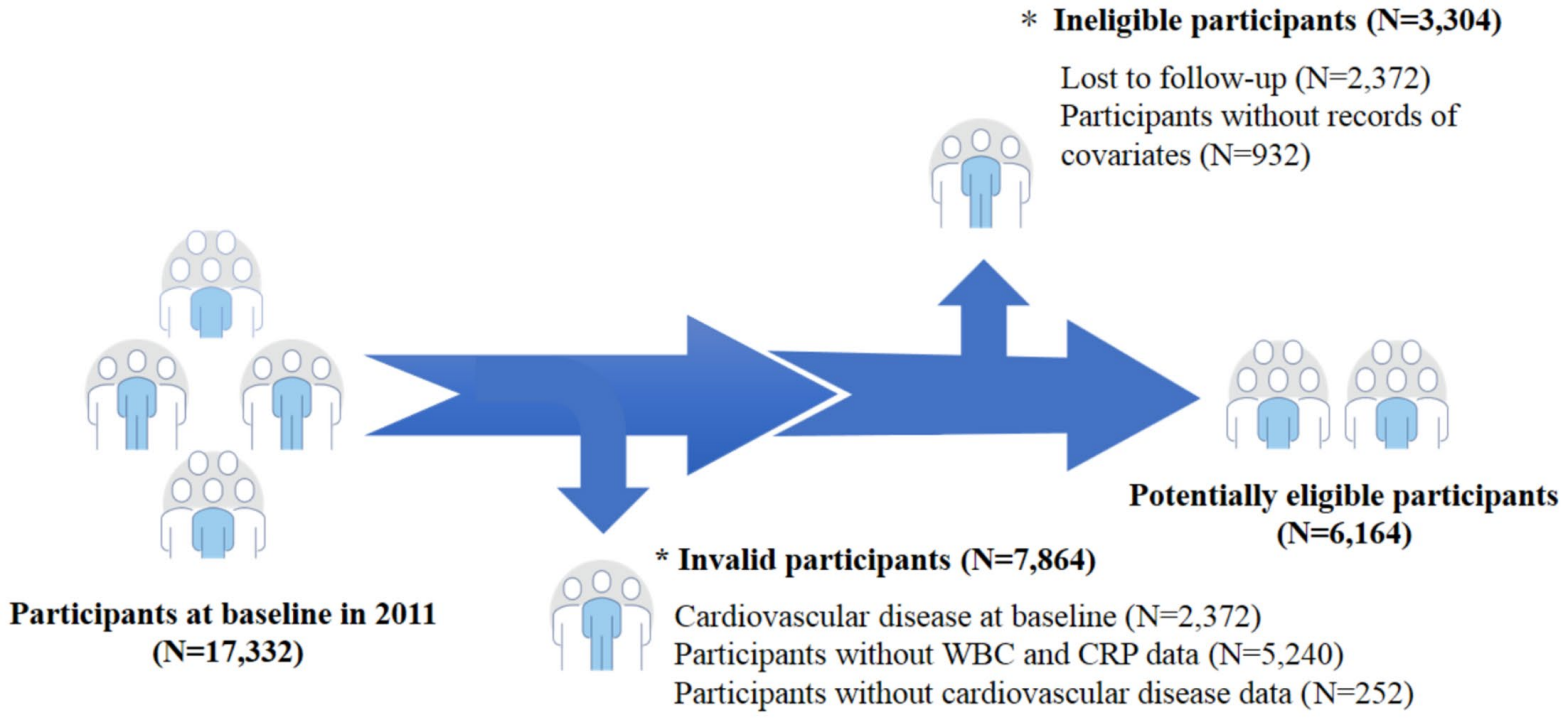
Flow chart of the study participant selection process.

A total of 17,332 participants aged ≥45 years were initially screened by us in the baseline survey. We excluded 7,864 participants without CVD data (*N* = 252), CVD at baseline (*N* = 2,372), and without WBC and CRP data (*N* = 5,240). In addition, we exclude 3,304 participants without records of covariates (*N* = 932) as well as those who were lost to follow-up during the follow-up process (*N* = 2,372). Finally, our statistical analysis included a total of 6,164 participants (Figure 1). The Peking University’s Ethical Review Committee approved our study and permitted the CHARLS, and all participants signed the informed consent forms to ensure the smooth progress of the research.

### Inflammatory score

After overnight fasting of the participants at baseline, venous blood samples were gathered. C-reaction protein was evaluated from the frozen plasma using immunoturbidimetric assay. White blood cell counts were analyzed and obtained on the analyzers provided in the laboratories after the samples of participants were collected (20). For each participant, Z-scores were calculated using their individual biomarker levels (X), the mean (M), and the standard deviation (SD) of the included participants, based on the following formula: Z-score = (X − M)/SD. Then, the calculation of the inflammatory score was made as the sum of the separate z-scores for CRP and WBC (11, 12).

### Healthy lifestyle score

The derivation of the Healthy lifestyle score (HLS) was based on the following lifestyle variables: 3 conventional factors [drinking status, body mass index (BMI), and smoking status] (21) and 1 emerging factor (sleep duration) (22). These factors derived from questionnaires and anthropometric measurements at baseline. Sleep duration was assessed using structured questionnaires. The adequate sleep duration was defined as night sleep time ≥ 7 h/day and night sleep time ≤ 8 h/day according to previous research (22). BMI was computed as weight (in kilograms) divided by the square of height (in meters). Trained staff members assessed the weight and height of the participants at the baseline using standardized equipment and techniques. Those who had a moderate BMI (18.5 ≤ BMI <25 kg/m^2^) were regarded as the healthy group (6). Information on smoking status and alcohol use was both gathered through a self-reported questionnaire. In the questionnaire survey, participants were required to record their smoking history and alcohol drinking habits. Participants scored 1 point for each healthy category defined based on previous studies and national guidelines. A healthy lifestyle was assessed as follows: non-smoking, non-drinking, 18.5 ≤ BMI < 25 kg/m^2^, and 7 ≤ sleep duration ≤8 h/day. The HLS score (ranging from 0 to 4 points) was computed as the cumulative sum of individual scores of the four lifestyle factors mentioned above, with higher scores indicating a more favorable lifestyle (22). We then categorized HLS into three categories, with 0–1 being the unfavorable, 2 the intermediate, and 3–4 the favorable.

### Assessment of CVD

In our study, the definition of CVD incidence (heart disease and stroke) was consistent with that of previous studies (1). During the face-to-face structured interviews in 2018, the trained investigators questioned the participants about whether they had been clinically diagnosed with CVD and the exact time of diagnosis. Participants were followed from baseline (2011) until the occurrence of stroke or cardiac events or the most recent survey (2018), whichever occurred first.

### Statistical analyses

To describe the baseline characteristics of the included participants, we used the median (interquartile range, IQR) when variables exhibited non-normal distribution, mean (SD) when variables displayed normal distribution, and number (proportion) for categorical variables. The ANOVA tests and chi-square tests were utilized to compare differences in the baseline characteristics of the participants. In addition, the Cox proportional hazard models were employed by us for assessing the associations of the inflammatory score and HLS with CVD risk and hazard ratios (HRs) and 95% confidence intervals (CIs) was calculated. The application of Schoenfeld residuals suggested no evidence of violation was found in the proportional hazard assumption (*p* > 0.05 for all). We adjusted for several potential confounders in these models according to the prior knowledge (23), including age (continuous; years), gender (men, women), education level (illiterate, primary school or below, middle school, high school or above), smoking status (yes, no), marital status (live with spouse, live without spouse), drinking status (yes, no), residence (urban, rural), BMI (kg/m^2^), and hypertension (yes, no).

To explore the modifying effects of overall lifestyle, we evaluated the relationships of inflammatory score with CVD risk according to overall lifestyle categories (tertiles of HLS). To evaluate the combined impacts of overall lifestyle and inflammatory score on the CVD risk, participants were categorized into nine groups based on inflammatory score (divided into low, medium, and high by tertiles) and overall lifestyle (divided into unhealthy, intermediate, and healthy by tertiles). The HR and 95%CI of CVD risk in different groups were calculated compared to those with the high healthy lifestyle score and low inflammatory score. We examined the modifying effect by incorporating interaction terms between inflammatory score and lifestyle. In addition, we computed the relative excess risk due to interaction (RERI) and their 95% CI (24), which were proposed as indicators of additive interaction in epidemiological research (25). The groups with low exposure to inflammatory score (below the median) and the healthiest lifestyle (high the median) were considered as references.

Multiple subgroup analyses were conducted by us to examine the relationship between inflammatory score and CVD based on gender (male or female), alcohol drink (yes or no), BMI (< 25 or ≥ 25 kg/m^2^), residence (rural or urban), and smoking status (yes or no). Moreover, additive and multiplicative interactions were also examined between inflammatory score and these stratifying factors.

We also performed sensitivity analyses to demonstrate the robustness of the findings. First, to address the possibility of reverse causality in the study, we excluded individuals with follow-up time ≤ 2 years. Second, to minimize potential confounding caused by ages, we excluded individuals aged ≥65 years. Finally, to assess the effect size for which unmeasured confounders made no statistical difference in the observed association between inflammation scores and CVD, we carry out an E - value analysis (26).

All statistical analyze were conducted using SAS version 9.4 (SAS Institute Inc.), and a level of two-sided *p* < 0.05 was considered statistically significant.

## Results

### Characteristics of the study participants

As shown in Table 1, among the 6,164 participants, a total of 761 incident CVD cases (12.46%) were detected during the 7 years follow-up period. And, 3,315 (54.29%) of the participants were women. The white blood cell counts and the levels of high-density lipoprotein cholesterol, low-density lipoprotein cholesterol total cholesterol, and triglyceride were higher in participants with higher inflammatory score (*p* < 0.05).

**Table 1:** Characteristics of the study population with various inflammatory score tertiles.

Variables	Inflammatory score			*p* valve
	Tertile 1	Tertile 2	Tertile 3	
Number of participants, *n*	2052	2053	2060	
Age (years)	57.00 (51.00, 63.00)	57.00 (51.00, 64.00)	57.00 (51.00, 64.00)	<0.05
BMI (kg/m^2^)	22.61 (20.69, 25.00)	23.13 (20.87, 25.67)	23.54 (21.11, 26.14)	0.30
Gender, *n* (%)				<0.05
Men	877 (42.74)	946 (46.08)	1,026 (49.83)	
Women	1,175 (57.26)	1,107 (53.92)	1,033 (50.17)	
Residence, *n* (%)				0.34
Rural	1,417 (69.05)	1,376 (67.02)	1,406 (68.29)	
Urban	635 (30.95)	677 (32.98)	653 (31.71)	
Marriage, *n* (%)				0.91
Married and living with spouse	1776 (86.55)	1759 (85.68)	1762 (85.58)	
Others	276 (13.45)	294 (14.32)	297 (14.42)	
Education, *n* (%)				0.25
Illiterate	603 (29.39)	583 (28.40)	576 (27.97)	
Primary school below	839 (40.89)	856 (41.70)	867 (42.11)	
Primary school	406 (19.79)	408 (19.87)	445 (21.61)	
Middle school or above	204 (9.93)	206 (10.03)	171 (8.31)	
Smoking status, *n* (%)				0.60
Yes	677 (32.99)	790 (38.48)	895 (43.47)	
No	1,375 (67.01)	1,263 (61.52)	1,164 (56.53)	
Drinking status, *n* (%)				0.07
Yes	519 (25.29)	565 (27.52)	549 (26.66)	
No	1,533 (74.71)	1,488 (72.48)	1,510 (73.34)	
Sleep duration, *n* (%)				<0.05
> 8 or < 7	873 (42.54)	847 (41.26)	848 (41.19)	
7–8	1,179 (57.46)	1,206 (58.74)	1,211 (58.81)	
Hypertension, *n* (%)				<0.05
Yes	639 (31.14)	740 (36.04)	816 (39.63)	
No	1,413 (68.86)	1,313 (63.96)	1,243 (60.37)	
Laboratory				
WBC (10^9^/L)	4.60 (4.10, 5.00)	6.00 (5.99, 6.40)	7.80 (7.20, 8.90)	<0.05
CRP (mg/dL)	0.64 (0.41, 1.12)	0.98 (0.54, 1.80)	1.75 (0.79, 4.10)	0.13
LDL-C (mg/dL)	111.73 (90.85, 132.60)	114.05 (93.56, 137.63)	117.14 (95.10, 140.34)	<0.05
HDL-C (mg/dL)	51.80 (42.53, 62.24)	49.87 (40.59, 60.70)	47.94 (39.04, 57.99)	<0.05
TC (mg/dL)	185.18 (164.30, 209.54)	190.98 (168.94, 215.34)	194.27 (169.33, 221.14)	<0.05
TG (mg/dL)	97.35 (69.47, 136.29)	105.32 (73.46, 155.76)	110.63 (79.65, 164.61)	<0.05

BMI, body mass index; CRP, C-reactive protein; HDL-C, high-density lipoprotein cholesterol; LDL-C, low-density lipoprotein cholesterol; TC, total cholesterol; TG, triglyceride; WBC, white blood cell. Values are numbers (percentages) for categorical variables and median (interquartile range) for continuous variables.

### Association between inflammatory score and incident CVD

Table 2 presented the positive correlation between the inflammatory score and the risk of CVD. After adjusting for multiple variables, we found that the highest quantile of the inflammatory score was correlated with the increased risk of CVD compared with the lowest (HR = 1.25; 95% CI = 1.04–1.49). Moreover, CVD risk increased 61% for every 10-unit increase in inflammation score (HR = 1.61; 95% CI = 1.13–2.30). Additionally, restricted cubic spline plot told us that there was no non-linear association between inflammatory score and CVD risk (*p* for non-linearity >0.05; Supplementary Figure S2). The results of the specialized association between WBC, CRP and CVD indicated that in Chinese ≥45 years, WBC and CRP were positively associated with CVD risk (WBC: HR = 1.24, 95% CI = 1.04–1.47; CRP: HR = 1.40, 95% CI = 1.17–1.68; Supplementary Table S1).

**Table 2:** Association between the inflammatory score and cardiovascular disease.

Characteristics	Tertiles of inflammatory score			*p* for trend*	Continuous**
	T1	T2	T3		
Cases/N	222/2052	254/2053	285/2059		761/6164
Model 1, HR (95% CI)	1.00 (Ref)	1.15 (0.96, 1.38)	1.30 (1.09, 1.55)	<0.05	1.69 (1.21, 2.36)
Model 2, HR (95% CI)	1.00 (Ref)	1.17 (0.97, 1.40)	1.32 (1.11, 1.58)	<0.05	1.72 (1.23, 2.41)
Model 3, HR (95% CI)	1.00 (Ref)	1.12 (0.94, 1.34)	1.25 (1.04, 1.49)	<0.05	1.61 (1.13, 2.30)

CI, confidence interval; HR, hazard ratio; Ref, reference; T, tertile. Model 1: Crude model; Model 2: Adjusted for age (years), gender (men, women); Model 3: Further adjusted for educational level (illiterate, primary school or below, middle school, high school or above), marital status (live with spouse, live without spouse), BMI (kg/m^2^), residence (urban, rural), smoking status (yes, no), drinking status (yes, no), and hypertension (no, yes). *p value for linear trend calculated from category median values. **Continuous intakes were calculated by per 10 unit increase.

### The association of HLS and incident CVD

Table 3 showed the negative correlation between HLS and the risk of CVD. When we adjusted for multiple variables, we found that participants in the healthy groups had lower CVD risk compared to those in the unhealthy group (HR = 0.74; 95% CI = 0.60–0.93). Moreover, for each 1-point increase in HLS, CVD risk decreased by 17% (HR = 0.83; 95% CI = 0.75–0.93). What’s more, regarding individual lifestyle factors, sufficient duration of sleep and moderate BMI were inversely correlated with CVD risk (Supplementary Table S2).

**Table 3:** Association between healthy lifestyle score and cardiovascular disease.

Characteristics	Healthy lifestyle score			*p* for trend*	Continuous**
	Unfavorable	Intermediate	Favorable		
Cases/N	150/1219	313/2257	298/2688		761/6164
Model 1, HR (95% CI)	1.00 (Ref)	1.13 (0.93, 1.37)	0.89 (0.73, 1.08)	0.08	0.92 (0.86, 0.99)
Model 2, HR (95% CI)	1.00 (Ref)	1.01 (0.82, 1.24)	0.74 (0.60, 0.93)	<0.05	0.86 (0.79, 0.93)
Model 3, HR (95% CI)	1.00 (Ref)	1.01 (0.82, 1.24)	0.74 (0.60, 0.93)	<0.05	0.83 (0.75, 0.93)

CI, confidence interval; HR, hazard ratio; Ref, reference. Model 1: Crude model; Model 2: Adjusted for age (years), gender (men, women); Model 3: Further adjusted for educational level (illiterate, primary school or below, middle school, high school or above), marital status (live with spouse, live without spouse), residence (urban, rural) and hypertension (no, yes). *p value for linear trend calculated from category median values. **Continuous intakes were calculated by per unit increase.

### Examination of effect modification by HLS

**Figure 2:**
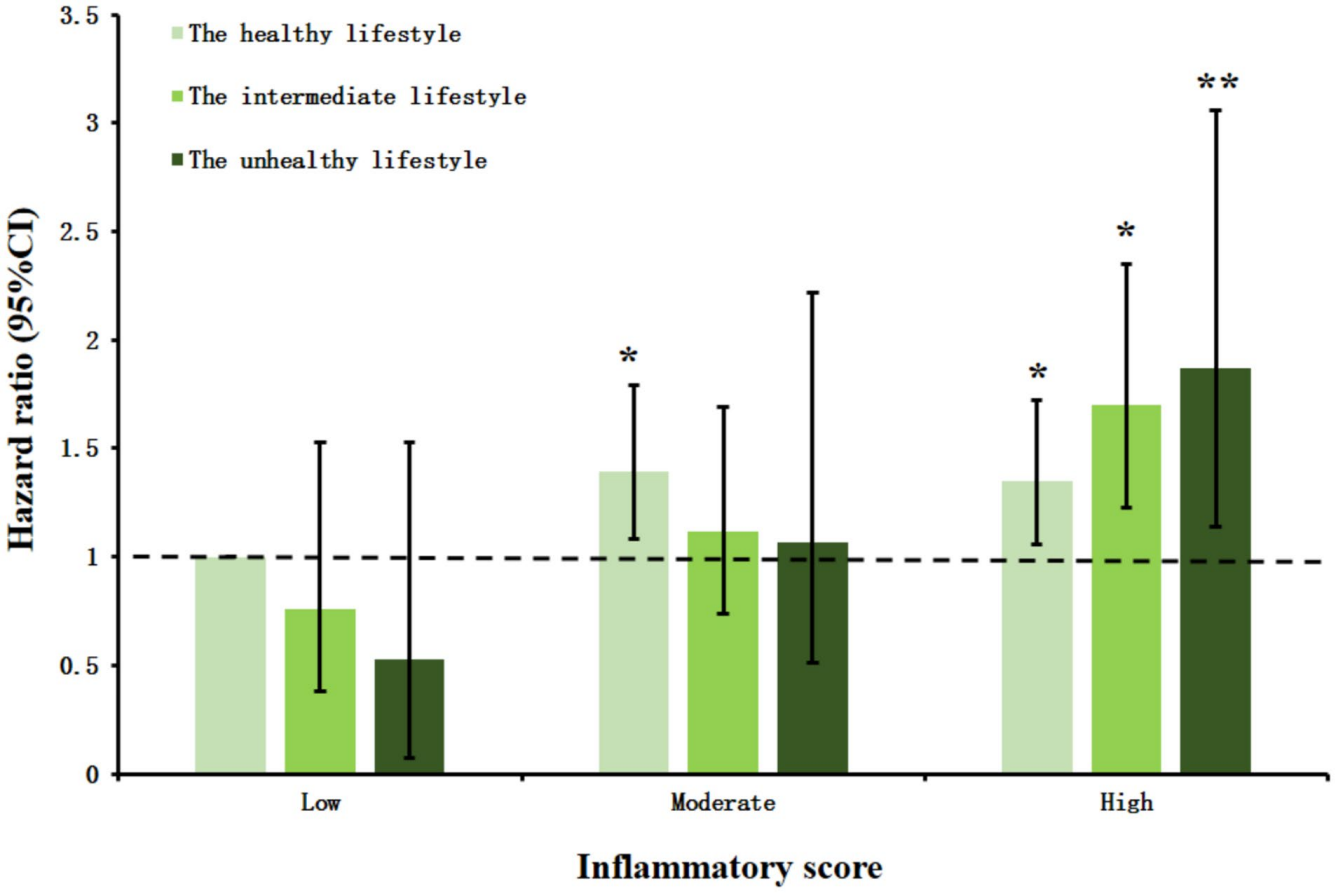
The joint effect between inflammatory score and healthy lifestyle score on the risk of cardiovascular disease incidence. HRs and 95% CIs were calculated with the use of the Cox proportional hazards regression model with adjustment for age, gender, education level, marital status, residence, hypertension. CI, confidence interval. *p < 0.05. **p < 0.01.

**Figure 3:**
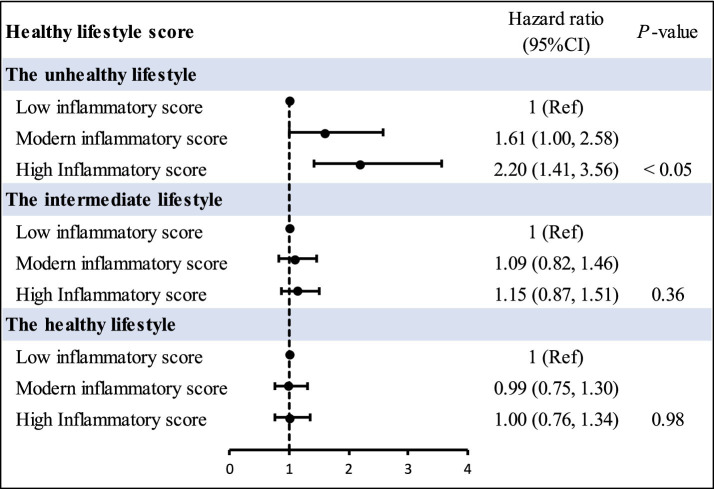
Incidence risk for cardiovascular disease according to inflammatory score and overall lifestyle categories. HRs and 95% CIs were obtained from Cox proportional hazards models, and all models were adjusted for age, body mass index, gender, alcohol drinking, cigarette smoking, education level, marital status, residence, hypertension. CI, confidence interval; Ref, reference.

Figure 2 revealed the associations of joint inflammatory score and HLS with CVD risk. The Joint effects of inflammatory score and HLS on the risk of CVD exhibited the person with the highest inflammatory score and the least healthy lifestyle had the higher CVD risk (HR = 1.87, 95% CI = 1.14–3.06). Moreover, in this study, we observed that a higher inflammation score was significantly associated with higher CVD risk in participants with an unhealthy lifestyle (HR = 2.20, 95% CI = 1.41–3.56), and this association was eliminated among those adhering to a healthier lifestyle (Figure 3). Additionally, we got that an negative association between overall healthy lifestyle and incident CVD persisted in participants with higher inflammation scores (Supplementary Figure S3). Notably, a multiplicative interaction of inflammation scores with the healthy lifestyle on the incidence of CVD was observed (Supplementary Table S3).

### Subgroup and sensitivity analysis

**Figure 4:**
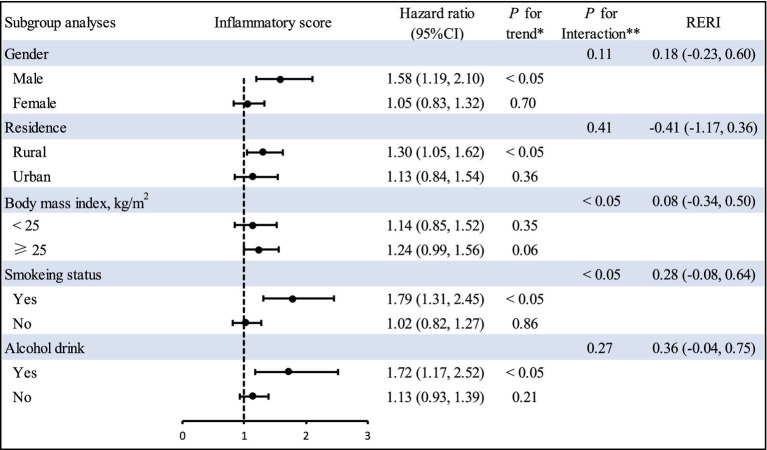
Subgroup analyses of inflammatory score and cardiovascular disease risk. The forest plot represents the hazard ratio of the comparison of the highest versus the lowest of inflammatory score. HRs and 95% CIs were adjusted for age, body mass index, gender, alcohol drinking, cigarette smoking, education level, marital status, residence, hypertension. *Indicates *P* for linear trend calculated from category median values. **Indicates *P* for interaction between strata and inflammatory score. *p* values are two-sided. CI, confidence interval; RERI, relative excess risk due to interaction.

We repeated our analyses in various dichotomous subgroups like gender (male or female), status of smoking (yes or no), region (rural or urban), BMI (<25 kg/m^2^ vs. ≥25 kg/m^2^), and alcohol drinking (yes or no). The majority of the results were consistent with our primary finding regarding the relationship between inflammatory score and CVD (Figure 4). Notably, a multiplicative interaction between smoke and inflammatory score on the risk of CVD was also observed. Additionally, we used multiple sensitivity analyses to confirm our findings. The relationship between inflammatory score and CVD risk remained robust after (I) excluding individuals with follow-up time ≤ 2 years (Supplementary Table S4), (II) excluding individuals aged 65 years or older (Supplementary Table S5), and (III) E-value analysis illustrated that a large number of confounding factors were required to explain this association (Supplementary Table S6).

## Discussion

In this population-based longitudinal cohort study, inflammatory score was observed to be associated with increased risk of CVD incidence, whereas adhering to an overall healthy lifestyle was significantly correlated with a decreased risk of CVD in middle-aged and older participants. Particularly, the deleterious impacts of inflammatory score on CVD appeared to be counteracted by an overall healthy lifestyle. In addition, a multiplicative interaction between inflammatory score and HLS for CVD risk was also detected.

To date, no epidemiological evidence have explored the association between inflammation score and CVD risk. Despite this, numerous studies have delved into the links between inflammatory markers and CVD risk. For example, a longitudinal cohort study of 15,828 individuals in Asian reported that WBC counts may be an independent predictive factor of CVD risk (OR = 2.45, 95% CI = 1.43–4.19) (27). Moreover, a large cohort study has shown that higher CRP levels may enhance the risk of cardiovascular events (8). Obviously, previous published studies have mainly analyzed on the association of individual inflammatory markers with CVD risk, which did not truly reflect the association between the level of inflammation in the body and CVD risk. Therefore, we use inflammation score to cleverly combine the two important indicators that mirror the inflammatory state of the organism (CRP and WBC), so as to more comprehensively reflect the inflammatory burden of the organism, which has a better prospect for clinical promotion than a single inflammation indicator. Nowadays, although the exact relationship between inflammation score and CVD has not been specifically elucidated, the findings between inflammation score and other health outcomes were analogous to those in our study. For instance, a longitudinal cohort study involving 3,401 patients with metabolic syndrome revealed that inflammation score was related to an increased risk of all-cause and cardiovascular death in participants with metabolic syndrome (11). Therefore, it is reasonable to expect that participants with higher inflammation scores would have an higher risk of CVD.

The findings of our study that a higher of HLS has a robust association with the risk of CVD was well aligned with previous studies (17, 18). Although lifestyle changes are considered a cost-effective intervention for preventing CVD, no studies have clearly assessed the potential impact of changing the aforementioned lifestyle factors on the relationship between inflammatory scores and the CVD risk. Therefore, our findings extended the strong evidence on the benefits of a healthy lifestyle by revealing that the positive correlation between higher levels of inflammation exposure and higher CVD risk was mitigated among participants who adhere to a healthy lifestyle. This result provided strong support for the public health efforts that emphasize a healthy lifestyle for anyone can reduce the burden of CVD. Moreover, a multiplicative interaction was observed between inflammation score and HLS on the CVD risk, indicating that a healthy lifestyle may lower the risk of CVD in participants exposed to higher levels of inflammation profile. Although no studies have examined the same association, there were some evidences indicating that adhering to an overall healthy lifestyle can decrease the degree of inflammation and oxidative stress within the body, consequently reducing the incidence of CVD (28–30). For example, a prospective study had illustrated that adhering to a healthy lifestyle was linked to lower levels of inflammation (31). Moreover, a cross-sectional study revealed that adhering to more healthy lifestyles was correlated with lower levels of inflammatory markers (32). An interventional study had also shown that adhering to an overall healthy lifestyle was able to decrease the burden of inflammation from exposure to systemic levels of inflammation (33). Given that low levels of inflammation may contribute to the development of chronic diseases and that maintaining a healthy lifestyle may reduce inflammation in the body, adopting an overall healthy lifestyle may reduce the risk of developing chronic diseases (32). In light of the global public health challenges posed by CVD and inflammation, enhancing our understanding of the risk factors and potential modifiers for CVD is crucial. According to public health policy and health promotion strategies, there is significant potential to optimize CVD prevention. This optimization can be achieved both through public initiatives and by encouraging individual health-promoting behaviors.

In the subgroup analysis, we observed that inflammation score may enhance the risk of CVD among smoking participants. In addition, we observed multiplicative interaction effects between inflammation score and smoking on the risk of CVD. This may be due to the fact that smoking is related to rising levels of several inflammatory markers, including C-reactive protein, tumor necrosis factor alpha, and interleukin-6 (34). Therefore, the presence of smoking may reinforce the role of inflammation score on the risk of CVD incidence. Additionally, non-smokers tend to have a better lifestyle than smokers. However, an unhealthy lifestyle may induce systemic inflammation (35). Therefore, inflammation score may not have harmful impact in non-smokers due to lower levels of systemic inflammation in non-smoking participants (3). These finding underscored the complex interaction of inflammation score and cigarette smoking on the incidence of CVD and underlined the significance of considering both when developing strategies to reduce CVD risk. In view of the insufficient number of participants in the subgroup analysis, we cannot exclude the possibility of chance findings. Further mechanism research will be required to confirm our findings.

Inflammation is one of the potential mechanisms of CVD (36). High levels of inflammation in the body will cause endothelial cells to be damaged during the inflammatory process, increase adhesion, promote the expression of adhesion factors, and allow white blood cells to cling to the blood vessel wall, which is significantly important for the formation of atherosclerosis (37). What’s more, atherosclerosis is the critical important cause of CVD (38). Fortunately, adhering to an overall healthy lifestyle confers cardiovascular benefits because it can reduce CVD risk factors in multiple dimensions. Overall improvements in lifestyle can attain cardiovascular benefits through reducing systemic inflammation (39, 40). Moreover, an unhealthy lifestyle might also contribute to the occurrence of CVD by enhancing thrombosis, oxidative stress, low-density lipoprotein cholesterol oxidation, and inflammation (34). Increased oxidative stress plays a critical role in the potential mechanisms that trigger cardiovascular dysfunction (34). Although these mechanisms for the effects of an overall lifestyle on the body’s inflammatory burden seem plausible, the exact causes and mechanisms require further exploration.

As far as we know, our study was the first to assess the relationship of inflammation score with CVD risk as well as explore potential modification impacts of overall lifestyle on this association. The main strengths lied in its incorporation of relatively large sample size, prospective design, high baseline participation, and consistent results across multiple sensitivity analyses. Our findings not only expanded the prospect and value of the inflammatory score in clinical application but also emphasized the significance of regulating the level of body inflammation to reduce the risk of CVD incidence. Of utmost importance is that our study indicated the inflammatory score represents a more preferable option when it comes to evaluating the risk of CVD incidence.

However, there were several potential limitations to be noted. First, due to ascertainment of lifestyle factors and physician-diagnosed condition was according to the self-reporting questionnaire, which was subjected to measurement errors and information bias. Nevertheless, face-to-face interviews performed by well-trained investigators were used to collect related information in this study, which might reduce these biases and improve accuracy. Second, lifestyle behaviors are subject to change over time. It is possible that misclassification may occur due to substantial alterations in lifestyle patterns during the follow-up period, and such misclassification might have exerted an impact on our estimates. In addition, due to questionnaire limitations, the HLS did not include diet and physical activity in this study, which may limit the ability to fully assess healthy lifestyles. Further research with more comprehensive healthy lifestyles are needed to confirm the present findings. Third, inflammatory score is sample-specific (11) and lifestyle factors distribution may vary across regions, ethnicities, and socioeconomic status (41, 42). Therefore, we should be cautious about the generalization of our findings. Further prospective research should evaluate the clear relationship between CVD and inflammatory score in various ethnic and populations. Finally, we despite our rigorous adjustment for major confounding variables, the possibility of residual confounding cannot be ruled out. However, E-value analysis of the present study showed that fairly large residual confounding factors was required to explain this association.

## Conclusion

Our findings first provided prospective evidence that the higher inflammatory score was associated with an increased CVD risk, whereas an overall healthy lifestyle was linked to a significantly decreased risk of CVD among middle-aged and elderly adults. Importantly, the adverse impacts of inflammatory score on CVD appeared to be offset by adhering to an overall healthy lifestyle. The results of our study may provide excellent implications for clinicians during the practice process, thereby presenting novel perspectives regarding comprehensive CVD management. Further large-scale, multi-center longitudinal research are needed to validate these associations.

## Data Availability

The raw data supporting the conclusions of this article will be made available by the authors, without undue reservation.
