# Supplementary material for: Association between inflammatory score, healthy lifestyle, and cardiovascular disease: a national cohort study
Source: Front Nutr. 2025 Feb 20;12:1534458. doi: 10.3389/fnut.2025.1534458 (PMC11882400; doi:10.3389/fnut.2025.1534458)
Supplement: Supplementary file 1 [file Data_Sheet_1.docx]

**Supplementary Table S1.** Association between the inflammation markers and cardiovascular disease.

| **Characteristics** | Tertiles of inflammation markers | | | *P* for trend* | Continuous** |
| --- | --- | --- | --- | --- | --- |
|  | T1 | T2 | T3 |  |  |
| **WBC** |  |  |  |  |  |
| Cases/N | 224/1999 | 240/2056 | 297/2109 |  | 761/6164 |
| Model 1, HR (95 % CI) | 1.00 (Ref) | 1.05 (0.87, 1.26) | 1.28 (1.07, 1.52) | < 0.05 | 2.90 (1.50, 5.68) |
| Model 2, HR (95 % CI) | 1.00 (Ref) | 1.06 (0.88, 1.27) | 1.30 (1.09, 1.54) | < 0.05 | 3.11 (1.60, 6.04) |
| Model 3, HR (95 % CI) | 1.00 (Ref) | 1.03 (0.86, 1.24) | 1.24 (1.04, 1.47) | < 0.05 | 2.63 (1.34, 5.15) |
| **CRP** |  |  |  |  |  |
| Cases/N | 204/2038 | 253/2060 | 304/2066 |  | 761/6164 |
| Model 1, HR (95 % CI) | 1.00 (Ref) | 1.24 (1.03, 1.49) | 1.51 (1.26, 1.80) | < 0.05 | 1.52 (0.89, 2.61) |
| Model 2, HR (95 % CI) | 1.00 (Ref) | 1.24 (1.03, 1.49) | 1.51 (1.27, 1.81) | < 0.05 | 1.54 (0.90, 2.65) |
| Model 3, HR (95 % CI) | 1.00 (Ref) | 1.21 (1.00, 1.45) | 1.40 (1.17, 1.68) | < 0.05 | 1.43 (0.81, 2.55) |

CI, confidence interval; CRP, C-reactive protein; HR, hazard ratio; Ref, reference; T, tertile; WBC, white blood cell.

Model 1: Crude model;

Model 2: Adjusted for age (years), gender (men, women);

Model 3: Further adjusted for educational level (illiterate, primary school or below, middle school, high school or above), marital status (live with spouse, live without spouse), BMI (kg/m^2^), residence (urban, rural), smoking status (yes, no), drinking status (yes, no), and hypertension (no, yes).

* *P* value for linear trend calculated from category median values.

** Continuous intakes were calculated by per 10 unit increase.

**Supplementary Table S2.** Association of lifestyle factors with cardiovascular disease.

| **Healthy lifestyle factors** | **HR (95% CI)** | ***P*-value** |
| --- | --- | --- |
| **Smoking status** |  |  |
| Ever/current smoking | 1.00 (Ref) |  |
| Non-smoking | 0.85 (0.69, 1.05) | 0.13 |
| **Alcohol use** |  |  |
| Ever/current drinking | 1.00 (Ref) |  |
| Non-drinking | 0.90 (0.75, 1.09) | 0.28 |
| **Sleep duration (hours/week)** |  |  |
| Insufficient sleep time | 1.00 (Ref) |  |
| Sufficient sleep time | 0.84 (0.72, 0.97) | < 0.05 |
| **BMI (kg/m^2^)** |  |  |
| Abnormal BMI | 1.00 (Ref) |  |
| Moderate BMI | 0.83 (0.72, 0.96) | < 0.05 |

BMI, body mass index; CI, confidence interval; HR, hazard ratio; Ref, reference.

Model adjusted for age (years), gender (men, women), educational level (illiterate, primary school or below, middle school, high school or above), marital status (live with spouse, live without spouse), BMI (kg/m^2^), residence (urban, rural), smoking status (yes, no), drinking status (yes, no), and hypertension (no, yes).

**Supplementary Table S3.** The additive and multiplicative interaction of inflammatory score and healthy lifestyle score on cardiovascular disease risk *.

| **Interactive items** | **Inflammatory score** |
| --- | --- |
| **Additive interaction** |  |
| RERI (95 % CI) | 0.26 (-0.07, 0.64) |
| **Multiplicative interaction** |  |
| *P*-value | < 0.05 |

CI, confidence interval; RERI, Relative excess risk due to interaction.

* RERI and 95 % CIs were calculated with the use of the Cox proportional hazards regression model with adjustment for age (years), gender (men, women), educational level (illiterate, primary school or below, middle school, high school or above), marital status (live with spouse, live without spouse), BMI (kg/m^2^), residence (urban, rural), smoking status (yes, no), drinking status (yes, no), and hypertension (no, yes).

**Supplementary Table S4.** Hazard ratios and 95% CI of the inflammatory score for cardiovascular disease among participants with the duration of follow-up more than two years.

| Characteristics | Tertiles of inflammatory score | | | *P* for trend* | Continuous** |
| --- | --- | --- | --- | --- | --- |
|  | T1 | T2 | T3 |  |  |
| Cases/N | 190/2017 | 221/2016 | 244/2025 |  |  |
| Model 1, HR (95 % CI) | 1.00 (Ref) | 1.18 (0.97, 1.43) | 1.30 (1.07, 1.57) | < 0.05 | 1.69 (1.18, 2.43) |
| Model 2, HR (95 % CI) | 1.00 (Ref) | 1.19 (0.98, 1.45) | 1.32 (1.09, 1.60) | < 0.05 | 1.73 (1.21, 2.49) |
| Model 3, HR (95 % CI) | 1.00 (Ref) | 1.15 (0.94, 1.40) | 1.25 (1.03, 1.51) | < 0.05 | 1.63 (1.11, 2.38) |

CI, confidence interval; HR, hazard ratio; Ref, reference; T, tertile.

Model 1: Crude model;

Model 2: Adjusted for age (years), gender (men, women);

Model 3: Further adjusted for educational level (illiterate, primary school or below, middle school, high school or above), marital status (live with spouse, live without spouse), BMI (kg/m^2^), residence (urban, rural), smoking status (yes, no), drinking status (yes, no), and hypertension (no, yes).

* *P* value for linear trend calculated from category median values.

** Continuous intakes were calculated by per 10 unit increase.

**Supplementary Table S5.** Hazard ratios and 95% CI of the inflammatory score for cardiovascular disease among participants aged 65 years or younger.

| Characteristics | Tertiles of inflammatory score | | | *P* for trend* | Continuous** |
| --- | --- | --- | --- | --- | --- |
|  | T1 | T2 | T3 |  |  |
| Cases/N | 152/1648 | 189/1648 | 228/1654 |  |  |
| Model 1, HR (95 % CI) | 1.00 (Ref) | 1.26 (1.02, 1.56) | 1.53 (1.25, 1.88) | < 0.05 | 1.94 (1.38, 2.73) |
| Model 2, HR (95 % CI) | 1.00 (Ref) | 1.28 (1.03, 1.58) | 1.58 (1.28, 1.94) | < 0.05 | 1.93 (1.38, 2.71) |
| Model 3, HR (95 % CI) | 1.00 (Ref) | 1.25 (1.01, 1.53) | 1.51 (1.23, 1.86) | < 0.05 | 1.94 (1.35, 2.78) |

CI, confidence interval; HR, hazard ratio; Ref, reference; T, tertile.

Model 1: Crude model;

Model 2: Adjusted for age (years), gender (men, women);

Model 3: Further adjusted for educational level (illiterate, primary school or below, middle school, high school or above), marital status (live with spouse, live without spouse), BMI (kg/m^2^), residence (urban, rural), smoking status (yes, no), drinking status (yes, no), and hypertension (no, yes).

* *P* value for linear trend calculated from category median values.

** Continuous intakes were calculated by per 10 unit increase.

**Supplementary Table S6.** Sensitivity analyses: E-values for the association between inflammatory score and risk of cardiovascular disease.

| Variables | E-values* for HR (and for CI) |
| --- | --- |
| Inflammatory score | 1.81 (1.24) |

CI, confidence interval; HR, odd ratio.

* E-value represents the minimum strength of association needed between an unmeasured confounder and both the exposure and the outcome to fully explain away the exposure-outcome association.


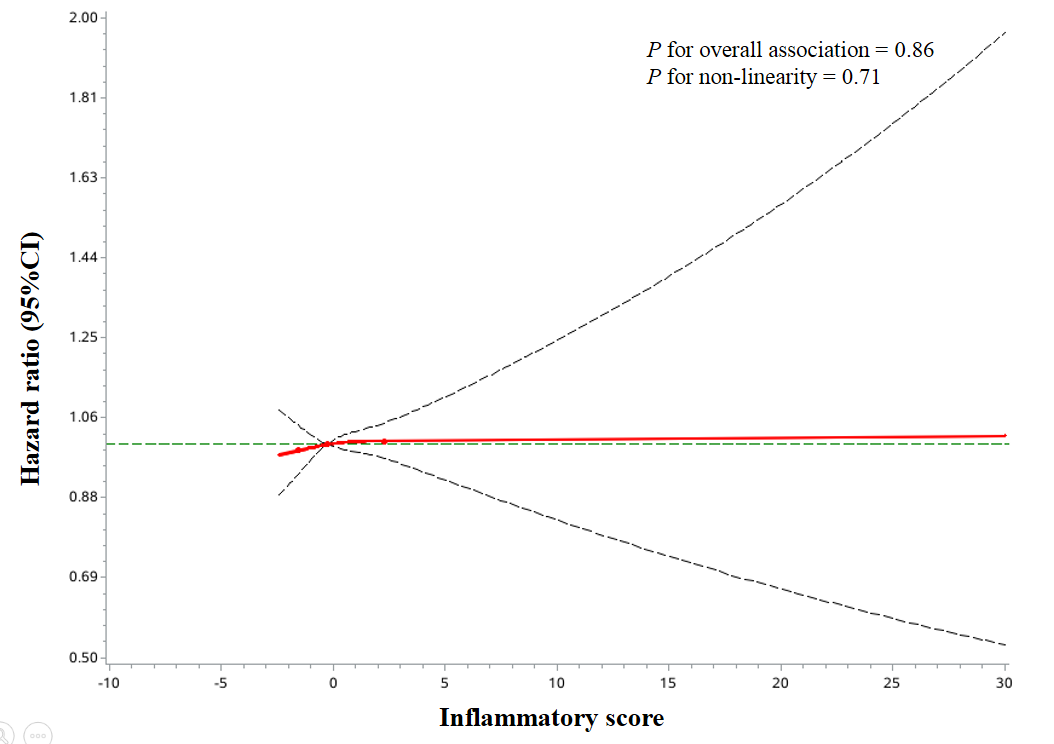


**Supplementary Figure S1.** The dose-response association between inflammatory score and cardiovascular disease. The association was adjusted for age, body mass index, gender, alcohol drinking, cigarette smoking, education level, marital status, residence, hypertension.

**Supplementary Figure S2.** Incidence risk for cardiovascular disease according to healthy lifestyle score and inflammatory score categories. CI, confidence interval; Ref, reference. HR (95% CI) were obtained from Cox proportional hazards models, and all models were adjusted for age, gender, education level, marital status, residence, hypertension.
